# Clinical outcome data for symptomatic breast cancer: the breast cancer clinical outcome measures (BCCOM) project

**DOI:** 10.1038/sj.bjc.6605155

**Published:** 2009-07-14

**Authors:** T Bates, O Kearins, I Monypenny, C Lagord, G Lawrence

**Affiliations:** 1 The Breast Unit, William Harvey Hospital, Ashford, Kent. TN24 OLZ, UK; 2 West Midlands Cancer Intelligence Unit, Quality Assurance Reference Centre, Public Health Building, University of Birmingham, Edgbaston, Birmingham, B15 2TT, UK; 3 University Hospital of Wales, Heath Park, Cardiff, CF14 4XW, UK

**Keywords:** breast neoplasm, data collection, clinical audit, mass screening, surgery, treatment outcome

## Abstract

**Background:**

Data collection for screen-detected breast cancer in the United Kingdom is fully funded, which has led to improvements in clinical practice. However, data on symptomatic cancer are deficient, and the aim of this project was to monitor the current practice.

**Methods:**

A data set was designed together with surrogate outcome measures to reflect best practice. Data from cancer registries initially required the consent of clinicians, but in the third year anonymised data were available.

**Results:**

The quality of data improved, but this varied by region and only a third of the cases were validated by clinicians. Regional variations in mastectomy rates were identified, and one-third of patients who underwent conservative surgery for the treatment invasive breast cancer were not recorded as receiving radiotherapy.

**Conclusion:**

National data are essential to ensure that all patients receive appropriate treatment for breast cancer, but variations still exist in the United Kingdom and further improvement in data capture is required.

The NHS Breast Screening Programme (NHSBSP), which was set up in 1988 based on the strength of the Forrest Report (Forrest, 1986), has had a number of important effects. At that time, the management of patients with breast cancer in the United Kingdom lay in the hands of general surgeons and, although many took special interest in the disease, the concepts of the breast care nurse and the multi-disciplinary team (MDT) were yet to be introduced in most hospitals. With the passage of time, the occasional operator came to accept the fact that the overall management of breast cancer required the attention of a dedicated team working out of a specialty breast unit, and the disciplines required for the screening process of specialist radiologists, surgeons and pathologists gradually took hold. However, the requirement for complete, accurate and timely data took longer to gain acceptance.

The collection of data on screen-detected breast cancer was funded from the outset by the NHSBSP, and has been facilitated by a single, breast screening computer system. In addition, the Regional Breast Screening QARCs (Quality Assurance Reference Centres) have been instrumental in providing good-quality data for audit (NHS Breast Screening Programme, 2008). The feedback on variations in practice at annual audit meetings organised both regionally and nationally, has identified outliers in clinical practice and, although peer pressure has been proved to be a slow process in establishing a consensus, it has been possible to show major changes in clinical practice over time (Sauven *et al*, 2003). The appointment of regional representatives for the screening programme led to the formation of the Breast Group of the British Association of Surgical Oncology (BASO), which in turn developed into the Association of Breast Surgery at BASO (ABS), and the presentation of NHSBSP/ABS audit data at the ABS Annual Meeting has become the main focal point for breast surgeons in the United Kingdom.

As the screening data became more robust, the lack of data for the majority of breast cancers that present symptomatically became more obvious, and with this recognition there was a growing concern that variations in the standard of care and sub-optimal practice might well be obscured. Since then, the lack of a national breast cancer database has been a limiting factor; although a BASO database was initially funded by Zeneca and later by the Department of Health, the software included all breast consultations and focussed on communication with the general practitioner rather than on systematic data collection. As a result, the database was not used widely and support was eventually withdrawn.

In response to these concerns, in 2000, the ABS initiated systematic data collection for symptomatic breast cancers and, with the support of those units with good data collection systems, achieved approximately one-third of the estimated national caseload. However, it became apparent with each year of this unfunded initiative that, as new units began submitting data, many collaborators failed to continue, often because of the withdrawal of funding for data managers. There was also a move by some acute hospital trusts to meet their responsibility to provide cancer waiting times data by extending the duties of established breast cancer data managers, which also had a negative effect. Over the same period, the ACGBI (Association of Coloproctology of Great Britain and Ireland) took a similar initiative to collect data on the management of colorectal cancer, and more recent attempts to collect data on oesophago-gastric and thyroid cancers by the respective professional associations (the Association of Upper Gastrointestinal Surgeons (AUGIS) and the British Association of Endocrine and Thyroid Surgeons (BAETS)), have suffered the same constraints, with retrieval rates of little more than a third of the national data.

Therefore, it became clear that requests made to individual clinicians or to units were not the right way forward, and in 2003 it was suggested that the data held by the regional cancer registries could be used to resolve the problem. Fears were expressed that data collection was less than complete in some registries, and it subsequently became apparent that permission for data release by individual clinicians and the requirement for anonymisation might be barriers to progress. It was at this stage that the Breast Cancer Clinical Outcome Measures (BCCOM) Project was established using a subset of the national breast cancer data set for maximising the ability of regional cancer registries to participate. As it is recognised that it takes some years before it becomes apparent whether variations in treatment lead to differences in disease-free and overall survival, a series of surrogate clinical outcome measures or ‘key performance indicators’ has been developed to monitor the extent to which best practice is followed.

## Methods

A breast cancer data set was designed after consultation with the ABS and the UKACR (UK Association of Cancer Registries). Data on all newly-diagnosed primary symptomatic breast cancers are obtained from the UK cancer registries and include basic demographic details, diagnostic information, tumour characteristics and the type of surgical and adjuvant treatment for each case. Male breast cancers are included, but screen-detected cases are excluded as far as possible. To reduce the contamination of symptomatic cases with screen-detected breast cancers, cases flagged by cancer registries as screen-detected breast cancers (as required in the national cancer registry peer review measures (Department of Health, 2005)) are excluded from the BCCOM data set. Cancer registries were asked to flag cases as having had a pre-operative diagnosis of breast cancer if the case record contained a cytology or core biopsy diagnosis that pre-dated the first therapeutic operation.

To validate the accuracy of data collection, cancer registries send the collected data to the concerned consultant breast surgeon. The surgeons in turn are asked to check the validity of data by comparing them with those held on local systems, to make amendments if necessary and to return the data without patient-identifiable details to the BCCOM Project team at the West Midlands Cancer Intelligence Unit (WMCIU). Surgeons may submit unchecked data if they do not have the necessary support mechanisms or if they are convinced that the quality of the data is high. Cases are not included if the surgeon attends less than six symptomatic cases in the year, chooses not to participate or is unknown.

From year 2 onwards, the initial protocol for data collection was modified to ensure compliance with Section 60 of the Health and Social Care Act 2001. It was observed that, although non-identifiable data were stored in the BCCOM central database, the flow of information at the beginning of the audit cycle, from cancer registry to surgeon for validation, was at an individual patient level. Therefore, the updated protocol requested that cancer registries obtain the written consent of individual consultant surgeons before releasing the data to the lead breast surgeon in each hospital. In year 2, all consultant breast surgeons, whether members of the ABS or not, were invited to participate in the BCCOM audit. The regional symptomatic surgical representatives contacted the lead breast surgeon in each hospital, seeking his (or her) help in collecting their colleagues' written consent to release data. In year 3, the process for data transfer from the cancer registries to the relevant consultant surgeon was altered such that for all registries apart from those in South West, Northern Ireland and Scotland, the data were distributed by the BCCOM team at the WMCIU. In addition, cancer registries provided the BCCOM team with data on all the breast cancers diagnosed in each region for that audit year (2004) so that an accurate denominator could be identified.

The data collected were analysed against the surrogate Clinical Outcome Measures devised by the BCCOM steering group (Table 1).

## Results

### Recruitment

**Figure 1:**
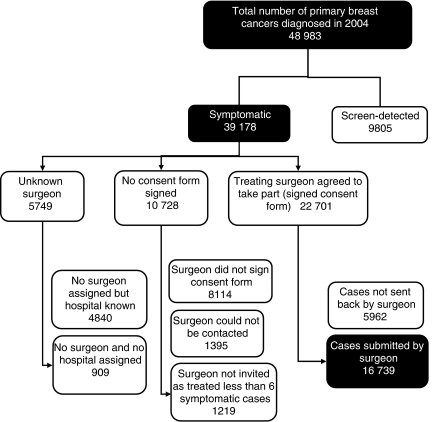
Total number of breast cancers recorded in BCCOM year 3 (cases diagnosed in 2004).

Table 2 shows participation levels in the BCCOM Project in each region and country. In year 2 (cases diagnosed in 2003), there was a 14% reduction in the total number of cases submitted (14 120 compared with 16 407) and very large reductions in some regions. These decreases are in part because of the more reliable exclusion of ineligible screen-detected cases in year 2, but mainly result from changes in the protocols for data collection in year 2, which required written consent from all surgeons before releasing the data of patients under their care to the lead surgeon in each hospital for validation. In year 3 (cases diagnosed in 2004), the UK cancer registries supplied the BCCOM team with data on all 48 983 diagnosed breast cancers. This provided a denominator of the total number of eligible cases with which participation could be compared (Table 3) and an estimate of the annual breast cancer burden in the United Kingdom could be made. Wales had the highest recruitment of cases at 94%, and the Thames Region, which has the highest number of surgeons and the most number of cases, had by far the lowest recruitment at 29%. Figure 1 shows that, in addition to the 1219 cases (3%), which were excluded in year 3 because the surgeon had treated fewer than six symptomatic cases, a further 21 220 symptomatic cases (54% of the total number of symptomatic cases identified by the cancer registries) could not be included either because the surgeon was non-compliant (15 471 cases) or unknown (5749 cases).

In year 3 (cases diagnosed in 2004), 16 611 female breast cancers were included and 128 breast cancers were detected in men. Slightly more breast cancers presented in the left breast (52 *vs* 48%). A total of 25% of cases were diagnosed in patients aged <50 years, 28% in those aged 50–64, 9% in those aged 65–69 and 37% in patients aged 70 or older.

### Screening flag

In year 3 (cases diagnosed in 2004), of the 48 983 breast cancers cases registered by cancer registries, 9805 (20%) were flagged as screen detected (Figure 1). From the NHSBSP/ABS audit of screen-detected cancers, it is known that 14 057 cases would have had a date of first offered appointment to screening in 2004, indicating that the cancer registries had accurately assigned only 70% of the screen-detected cases. Those regions that did not have the robust communications between cancer registries and breast screening QA reference centres, which are required to flag screen-detected breast cancers accurately, tended to have the highest rates of non-invasive breast cancers (up to 10% in year 1) and the greatest proportion of cases in the then screening age group (50–64 years) included in their BCCOM cohorts. The proportion of non-invasive breast cancers decreased from 6.3% in year 1 to 5.8% in year 3, but this rate is still higher than that expected from the literature, which suggests that only 3% of non-invasive breast cancers present symptomatically (Blamey *et al*, 2000) compared with 21% (including micro-invasion) of screen-detected cases (NHS Breast Screening Programme, 2008). This provides surrogate evidence of continuing contamination by screen-detected breast cancers in some regions. The recent requirement of the national cancer registry peer review measures for registries to obtain details of screen-detected breast cancers from breast screening QA reference centres has greatly improved the situation compared with that of 2003, and it is hoped that in year 4 (cases diagnosed in 2005), all registries will have correctly identified their screen-detected cases.

### Histological type

Of the 47 266 breast cancer cases that were submitted to BCCOM during years 1–3, invasive ductal carcinoma was the most common histological type (68%), followed by invasive lobular carcinoma (12%), ductal carcinoma *in situ* (5%), mixed invasive carcinoma (5%), mucinous carcinoma (2%) and tubular carcinoma (1%). These proportions will probably change slightly when all screen-detected cases have been eliminated, but they illustrate how the audit could provide a source of a relatively large number of rarer tumours for research.

### Nodal status

Of the breast cancer cases submitted in year 3 (cases diagnosed in 2004), 31.8% were lymph node positive, 34.3% were lymph node negative and 33.9% had unknown nodal status (Table 4). For surgically treated cases, 40.5% were lymph node positive, and the proportion with unknown lymph node status was 14.4%. The relatively high proportion of surgically treated cases with unknown lymph node status may be because of the fact that some cancer registries do not record data on lymph node status and tumour size for patients who receive neoadjuvant chemotherapy or radiotherapy. This is because the use of such data to determine the Nottingham Prognostic Index (NPI) (Haybittle *et al*, 1982) or the pathological TNM stage at diagnosis could result in an inaccurate under-staging of the cancer. Recording of the axillary node status increased during years 2–3 of the audit for all age groups, but was higher in those under the age of 50 years (89%) than in those aged over 80 (72%), largely because the latter group are less likely to undergo surgery.

### Tumour size

In year 3, for 31.4% of the cancers included in the cohort, the maximum diameter of the invasive tumour component was <20 mm, and for 24.6% of cases the invasive size was unknown. For surgically treated cases, the invasive tumour size was unknown for only 7% of cancers. In most of the latter cases, the invasive size at diagnosis was not recorded either because the patient had received neoadjuvant treatment, which may have reduced the original size at diagnosis, or because the tumour was removed in several pieces from more than one operation.

### Tumour grade

**Figure 2:**
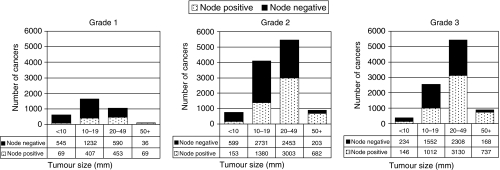
Variation in lymph node status with tumour grade and size for tumours included in BCCOM years 1–3.

In year 3, 12.0% of invasive cancers were classified as grade 1, 41.0% were grade 2 and 33.2% were grade 3. For surgically treated cases, these proportions were 12.8, 43.3 and 37.9% respectively. The grade was unknown for 13.9% of all cases, but this decreased to 6.0% for surgically treated cases. Pathologists are reluctant to report the grade of the tumour after neoadjuvant treatment, which may partly explain the latter shortfall. There was little variation in tumour grade over the 3 years of the study. There was a clear association between nodal status, tumour grade and size; with grade 1 cancers being smaller and more likely to be node negative (Figure 2).

### Nottingham prognostic index

**Figure 3:**
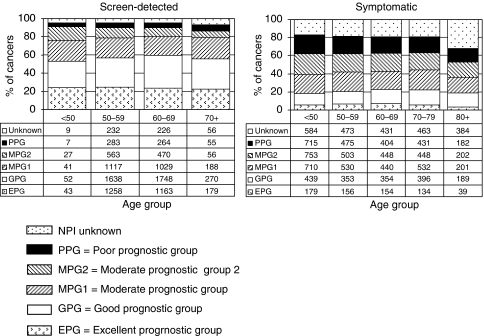
Variation in Nottingham Prognostic Index (NPI) with age group for breast cancers diagnosed in 2004. Sources: screen-detected breast cancers included in the NHSBSP/ABS annual audits (2003–2004 and 2004–2005) of screen-detected breast cancer; symptomatic breast cancers included in BCCOM year 3.

In year 3, the NPI score could be calculated for 80% of the surgically treated invasive breast cancers. The NPI could not be calculated in 20% of the cases because of missing grade (6%), size (7%) and/or nodal status (14%). Nodal status was not available in 28% of patients over the age of 80 years. Among those cases with a known NPI, 51% were early breast cancers with an NPI score of below 4.4 and were categorised into the Excellent Prognostic Group (EPG), Good Prognostic Group (GPG) or Moderate Prognostic Group 1 (MPG1). Overall, 49% were categorised into the MPG2 or Poor Prognostic Group (PPG) (Blamey *et al*, 2007). These data are in marked contrast to screen-detected breast cancers. In the NHSBSP/ABS audit of screen-detected breast cancers that were diagnosed in 2004, 83% of cases had an NPI score of below 4.4 (24% in the EPG, 36% in the GPG and 22% in the MPG1), 11% were in the MPG2 and 6% in the PPG. The variation in NPI with age at diagnosis for surgically treated screen-detected and symptomatic breast cancers is shown in Figure 3.

### Surrogate clinical outcome measures

**Figure 4:**
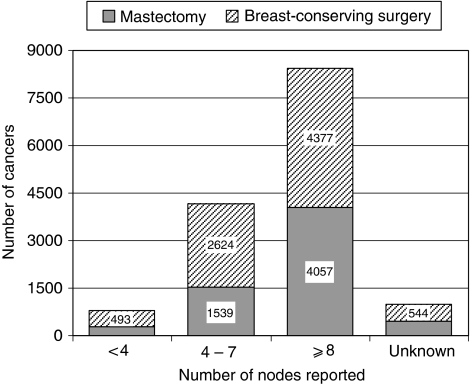
Variation in the number of nodes reported in node-negative patients in BCCOM years 1–3 who received breast-conserving surgery or mastectomy.

The surrogate clinical outcome measures proposed by the BCCOM Project team are shown in Table 1. The number of cases treated in each breast unit cannot be calculated from BCCOM data as not all surgeons agreed to participate in the audit. Pre-operative diagnosis rates varied between 12% in Scotland and 87% in the West Midlands and were 40% or less in five regions. The NHSBSP/ABS audit of screen-detected breast cancer has shown an improvement in pre-operative diagnosis from 63% between 1996 and 1997 to 94% between 2006 and 2007 (NHS Breast Screening Programme, 2008). Reliable pre-operative diagnosis data were only available from at most three cancer registries, because many of them record data only from pathology reports for resection specimens and do not record details from any preceding cytology or core biopsy reports. The numbers of nodes reported in what proved to be a negative sample are shown in Figure 4. In those patients treated with breast-conserving surgery, the majority with negative axillae had eight or more nodes reported.

### Surgical treatment

**Figure 5:**
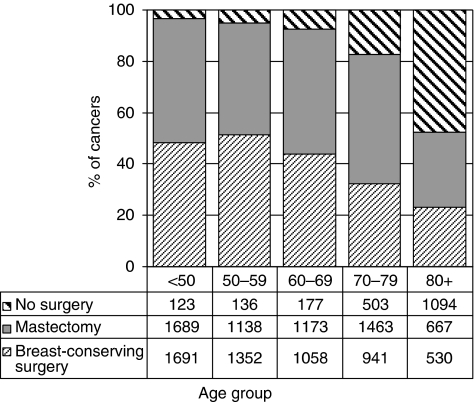
Variation in surgical treatment with age at diagnosis in BCCOM year 3 (invasive cancers diagnosed in 2004).

**Figure 6:**
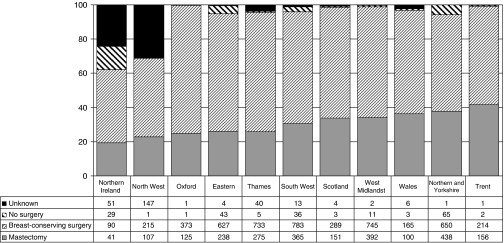
Variation with region and Celtic country in the operations recorded for patients with small invasive breast cancers (invasive diameter <15 mm) in BCCOM year 3 (cancers diagnosed in 2004).

Variations in the treatment of invasive cancers with age at diagnosis in year 3 are shown in Figure 5. The proportion of women not receiving surgery increased with age from 3.5% in women aged <50 years to 47.7% in women aged 80 or more. The proportion receiving breast-conserving surgery decreased with age from 51.4% in women aged <65 years to 41.9% in women aged 65 or more. For surgically treated cases, in each region, the breast-conserving surgery rate was higher in younger patients, but this difference between age groups was most marked in Oxford (58 *vs* 43%) and in Wales (54 *vs* 26%). The proportion of cases receiving breast-conserving surgery was lower than the UK average of 47.6% in Trent, Northern Ireland and Northern and Yorkshire and was higher than the UK average in the Thames Region. Figure 6 shows the regional variation in the operation types recorded for invasive breast cancers with a diameter <15 mm. At 42%, the Trent Region had the highest mastectomy rate for this group of small tumours, and Northern Ireland and the North West Region the lowest (19 and 23% respectively). However, as the proportion of cases with unknown operation type was high in these areas, care should be taken in the interpretation of these reported patterns of care.

### Adjuvant treatment

**Figure 7:**
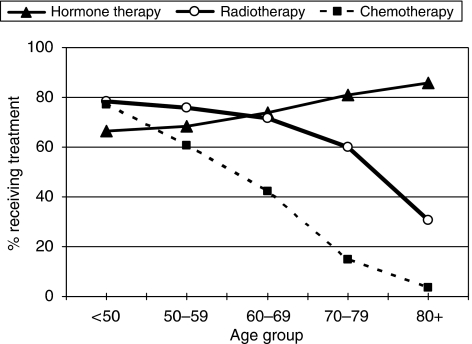
Variation in adjuvant treatment with age at diagnosis for BCCOM years 1–3. Cases for which it was not known whether a patient had received treatment have been excluded. In the elderly patients, hormone therapy was sometimes the sole primary treatment.

Figure 7 shows, for all breast cancer patients with known adjuvant therapy included in BCCOM years 1–3, how the proportions of cases receiving adjuvant radiotherapy, chemotherapy and hormone therapy vary with age at diagnosis. The recorded use of hormone therapy increases with age, with 85.6% of patients aged 80 years and more receiving hormone therapy compared with 66.4% of patients aged <50 years. This older age group is less likely to receive surgical intervention, and as such hormone therapy may be the only form of active treatment provided. In contrast, the recorded use of radiotherapy decreases with increasing age. In total, 78.3% of the patients aged <50 years received radiotherapy compared with 30.6% of patients aged over 80. The effect of age on recorded treatment modality is most marked for chemotherapy, where 77.2% of patients aged <50 received chemotherapy, but only 21.9% of patients aged 65–79 and 16% of patients aged 65 and more.

In the 3-year period between 2002 and 2004, radiotherapy was recorded as having been received by 68.7% of the 16 487 patients included in the audit who were treated with conservative surgery. A total of 1126 cases (6.8%) were recorded as not having received radiotherapy, but for a further 4029 cases (24.4%), it was not known whether radiotherapy was given. Fewer elderly patients were recorded as having undergone radiotherapy after conservative surgery, with the proportion known to have received radiotherapy decreasing from 70% in patients aged under 50 years to 43% in those aged 80 and above.

**Figure 8:**
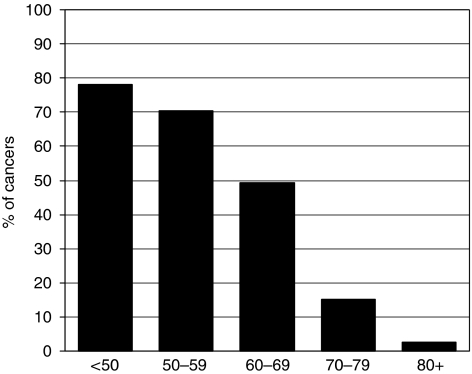
Variation with age at diagnosis in the proportion of node-positive breast cancer patients in BCCOM years 1–3 recorded as having received chemotherapy. Cases or which it was not known whether a patient had received chemotherapy have been excluded.

In the 3-year period of 2002–2004, chemotherapy was recorded as having been received by 53% of the 13 100 patients with invasive breast cancer who were node positive (Figure 8). A total of 2630 cases (20.1%) were recorded as not having received chemotherapy and for a further 3524 cases (26.9%), it was not known whether chemotherapy was given. In node-positive patients under the age of 70 years, the proportion known to have received adjuvant chemotherapy was 68% compared with only 12% in those aged 70 or more.

Of the cases with known hormone treatment that were receptor positive (oestrogen receptor (ER) positive and/or progesterone receptor), 11% (1241 cases) did not receive any form of hormone treatment. For 16% (2418 cases) of the receptor-positive invasive cancers, it was not known whether hormone treatment was given. Only 3961 cases were receptor negative and of these, 9% (367 cases) were known to have been prescribed hormone therapy. Of the 5112 invasive breast cancer cases who did not undergo surgery, 3106 (61%) were recorded as having received hormone therapy, but only 2176 (43%) had known ER status. It would be anticipated that the majority of these mostly elderly patients, who did not undergo an operation, would have had strong contraindications to surgery and would have been treated with hormonal therapy. Unfortunately, for all cases for which hormone therapy data are recorded, tamoxifen is not distinguished from aromatase inhibitors and switches are not identified.

## Discussion

Participation by breast surgeons in the BCCOM Project is not mandatory, but it is strongly encouraged by their professional body, the ABS. Previous experience with the NHSBSP/ABS audit of screen-detected breast cancers has shown that a regular audit of surgical practice improves standards and highlights outliers, in which local protocols are not in keeping with the accepted best practice (Sauven *et al*, 2003). The regional symptomatic representatives of ABS are encouraged to review participation in their own areas and to identify ways in which this could be improved. Although progress in data collection has been improved by central notification of surgeons in most regions, the data in Figure 1 underline the continuing difficulty in depending on the voluntary and active participation of individual surgeons in the submission and validation of data. The surgeon does not own the data, and although their written permission for the release of patient details under their care has been a prerequisite of the BCCOM audit to date, it seems clear that the collection of cases will not approach completeness on this basis. Furthermore, patients are increasingly managed by a MDT rather than by an individual consultant surgeon, who will be involved in the initial management plan but who may have little or no responsibility for the subsequent treatment.

At a national level, cancer registry data are now matched to data held in national data sets, such as Hospital Episode Statistics (HES). From those cancer registries, which routinely compare their data with those on HES, it has become apparent that the latter can provide useful information on operations for which the pathology reports may not have been transferred to or accessed by cancer registries because no malignancy is reported. These include additional operations to remove nodes that are negative for tumour deposits and repeat operations on the breast, such as delayed reconstruction, which have a benign pathological outcome. Most importantly, matching cancer registration and HES data also allows the identification of surgeons and hospitals for each type of treatment if these data have not been collected by the cancer registry, thus increasing the number of cases that can be returned to surgeons for checking.

It has been possible to derive the surrogate outcome measures proposed by the BCCOM Project team for a high proportion of the symptomatic breast cancers included in the audit. The surrogate outcome measures developed to date are restricted, to an extent, by the common data items available from all cancer registries. As yet, quality-of-life data and/or patient-reported outcome measures have been collected on a research basis only, but it is clear that they should become part of the standard outcome measures in the future. The inclusion of reconstruction after mastectomy as a key performance indicator should also be considered, and it is hoped that the inclusion of a surrogate outcome measure for this area will be possible once HES data are obtained for all breast cancer cases treated in England.

Regional variations in surgical practice, especially with respect to mastectomy rates, have been highlighted in the BCCOM audit, but variations in individual clinical practice are more difficult to identify as data were collected by the hospital or unit (Moritz *et al*, 1997). The reasons for regional variations are unclear, but mastectomy rates tend to be higher in rural areas and this association is not confined to the United Kingdom (Craft *et al*, 1997; Gort *et al*, 2007). The data for 2002–2004 indicate that patients with lymph node-negative disease had a large number of nodes removed even when the surgical procedure was conservative (Figure 4). This time period reflects practice before as well as including the wide-scale introduction of sentinel lymph node biopsy (SLNB) for which the audit protocol required a nodal clearance for all patients undergoing SNLB, and future data should show a change in this practice. Variations in practice style by individual surgeons are well recognised (Craft *et al*, 1997; Hawley *et al*, 2006), but in breast cancer, any consequent variation in patient outcomes such as recurrence rates or overall survival rates may take many years to become apparent (Purushotham *et al*, 2001). It is for this reason that surrogate clinical outcome measures have been proposed to reflect best practice, in order that publication of the data may try to persuade outliers to change their practice.

The place of radiotherapy after conservative surgery for invasive breast cancer is well-established (Clarke *et al*, 2005), and yet there is evidence that this treatment has been under-used. There may occasionally be a good reason not to give post-operative radiotherapy but, if the BCCOM data are correct, a third of such patients did not undergo prophylactic treatment and a third of these would be expected to develop local recurrence. The indications for radiotherapy for patients with *in situ* breast cancer are less well defined, but current variations in practice are not always based on the available evidence (Dodwell *et al*, 2007). There is also concern that 20% of patients with node-positive disease under the age of 50 years did not receive adjuvant chemotherapy (Figure 7). There is now a requirement that the treatment of all patients with breast cancer should be considered at a multi-disciplinary meeting, and any failure to consider an appropriate adjuvant treatment should be a thing of the past. Reflection on performance data such as those provided by audits such as BCCOM should assist local breast teams in identifying any non-compliance with national practice in their protocols and for facilitating the targeting of areas requiring modifications to make them consistent with best clinical practice.

## Figures and Tables

**Table 1:** Surrogate clinical outcome measures for breast cancer proposed by the BCCOM Project team

**Proposed surrogate clinical outcome measures**
1. Number and proportion of breast cancers for which complete information is received
2. Number of symptomatic and screen-detected breast cancers treated in a hospital per annum
3. Number and proportion of breast cancers for which there is a pre-operative diagnosis
4. Number and proportion of breast cancers given medical treatment only
5. Number and proportion of breast cancers treated surgically
6. Mastectomy rate by breast size: <15; ⩾15 and ⩽20; >20 and ⩽35; >35 and ⩽50; >50 mm invasive diameter
7. Number and proportion of invasive breast cancers for which nodal status is known
8. Number and proportion of histologically node-negative invasive breast cancers for which more than seven nodes were harvested
9. Number and proportion of invasive breast cancers treated by breast-conserving surgery and receiving radiotherapy
10. Number and proportion of node-positive patients with invasive breast cancers, aged <60 years, receiving chemotherapy
11. Number and proportion of patients with ER-positive invasive breast cancers, receiving hormone therapy

BCCOM=breast cancer clinical outcome measure; ER=oestrogen receptor.

**Table 2:** Participation by regions and Celtic countries in years 1, 2 and 3 of the BCCOM project

	**Diagnosis year**				
	**2002**	**2003**	**2004**	**2002–2004**	
**Region or Celtic country**	**BCCOM year 1**	**BCCOM year 2**	**BCCOM year 3**	**Total**	**% eligible cases year 3**
Eastern	1691	997	1507	4195	65
North West	1091	524	1397	3012	41
Northern and Yorkshire	2419	2029	1910	6358	52
Northern Ireland	640	367	432	1439	45
Oxford	1341	1442	899	3682	62
Scotland	934	181	1836	2951	49
South West	3253	1001	2234	6488	54
Thames	1750	2709	1530	5989	29
Trent	408	1588	1453	3449	52
Wales	351	952	1201	2504	94
West Midlands	2529	2330	2340	7199	77
Total	16 407	14 120	16 739	47 266	52

BCCOM=breast cancer clinical outcome measure.

**Table 3:** Participation by surgeons in year 3 of the BCCOM project (cases diagnosed in 2004)

	**Eligible surgeons^a^**			**Eligible surgeons who submitted data**			**Take up BCCOM year 3**	
**Region or Celtic country**	**Number of surgeons**	**Number of cases**	**Average, cases/surgeon**	**Number of surgeons**	**Number of cases**	**Average, cases/surgeon**	**% of eligible surgeons**	**% of eligible cases**
Eastern	42	2314	55	15	1507	100	35.7	65.1
North West	66	3442	52	20	1397	70	30.3	40.6
Northern and Yorkshire	55	3692	67	25	1910	76	45.5	51.7
Northern Ireland	16	962	60	6	432	72	37.5	44.9
Oxford	18	1447	80	12	899	75	66.7	62.1
Scotland	46	3767	82	30	1836	61	65.2	48.7
South West	56	4121	74	27	2234	83	48.2	54.2
Thames	77	5283	69	18	1530	85	23.4	29.0
Trent	35	2782	79	15	1453	97	42.9	52.2
Wales	28	1276	46	18	1201	67	64.3	94.1
West Midlands	49	3027	62	35	2340	67	71.4	77.3
Total	488	32 113	66	221	16 739	76	45.3	52.1

BCCOM=breast cancer clinical outcome measure.

a Surgeons were eligible if they treated 6 or more symptomatic breast cancer cases in 2004.

**Table 4:** Characteristics of the invasive breast cancers included in year 3 of the BCCOM Project (cases diagnosed in 2004)

	**All invasive (15** **540)**			**Surgically treated only (11** **725)**		
**Invasive breast cancers diagnosed in 2004**	**No. of cases**	**%**	**% (when known)**	**No. of cases**	**%**	**% (when known)**
*Nodal status*						
Positive	4941	31.8	48	4754	40.5	47
Negative	5332	34.3	52	5287	45.1	53
Unknown	5267	33.9	NA	1684	14.4	NA
						
*Grade*						
I	1862	12.0	14	1501	12.8	14
II	6371	41.0	48	5073	43.3	46
III	5152	33.2	38	4449	37.9	40
Unknown	2155	13.9	NA	702	6.0	NA
						
*Invasive size*						
<15 mm	2544	16.4	22	2360	20.1	22
15–<20 mm	2340	15.1	20	2220	18.9	20
20–<50 mm	5862	37.7	50	5472	46.7	50
50+mm	968	6.2	8	849	7.2	8
Unknown	3826	24.6	NA	824	7.0	NA
						
*NPI*						
EPG+GPG+MPG1	4896	31.5	51	4816	41.1	51
MPG2 + PPG	4673	30.1	49	4567	39.0	49
Unknown	5971	38.4	NA	2342	20.0	NA
						
*Surgery*						
Breast-conserving surgery	5583	35.9	41	5583	47.6	NA
Mastectomy	6142	39.5	45	6142	52.4	NA
No surgery	2034	13.1	15	NA	NA	NA
Unknown	1781	11.5	NA	NA	NA	NA

BCCOM=breast cancer clinical outcome measure; EPG= Excellent Prognostic Group; GPG= Good Prognostic Group; MPG1= Moderate Prognostic Group 1; MPG2= Moderate Prognostic Group 2; NPI= Nottingham Prognostic Index; PPG= Poor Prognostic Group.
