# Clinical outcome data for symptomatic breast cancer: the Breast Cancer Clinical Outcome Measures (BCCOM) project

**DOI:** 10.1038/sj.bjc.6605294

**Published:** 2009-09-08

**Authors:** T Bates, O Kearins, I Monypenny, C Lagord, G Lawrence

**Correction to**: *British Journal of Cancer* (2009) **101**, 395–402; doi:10.1038/sj.bjc.6605155

Upon publication of this paper in Volume 101, the authors noticed an error in the legend of Figure 6. The correct legend with changes highlighted in bold is reproduced below, along with the original figure.

## Figures and Tables

**Figure 6:**
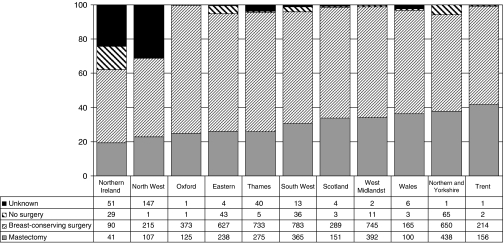
Variation with region and Celtic country in the operations recorded for patients with small invasive breast cancers (invasive diameter less than 15 mm) in BCCOM **Years 1**–**3** (cancers diagnosed in **2002**–**2004**).

